# [Corrigendum] Anti-carcinogenic properties of omeprazole against human colon cancer cells and azoxymethane-induced colonic aberrant crypt foci formation in rats

**DOI:** 10.3892/ijo.2024.5709

**Published:** 2024-11-25

**Authors:** Jagan M.R. Patlolla, Yuting Zhang, Qian Li, Vernon E. Steele, Chinthalapally V. Rao

Int J Oncol 40: 170-175, 2012; DOI: 10.3892/ijo.2011.1214

Following the publication of the above article, an interested reader drew to the authors' attention that certain of the *in vitro* image panels shown in Fig. 3B (featuring the effects of adding five different concentrations of omeprazole on acridine orange/ethidium bromide-stained HCA-7 cells) and Fig. 4 (showing western blotting experiments) on p. 173 and 174 respectively contained overlapping data panels, where results that were intended to represent the results of differently performed experiments had apparently been derived from the same original sources. Specifically, the image panels for 0 and 50 *μ*M/ml omeprazole (Fig. 3Ba and Bb), and 100 and 200 *μ*M/ml omeprazole (Fig. 3Bc and Bd), in Fig. 3B were strikingly similar; and the bands shown for p21 and cyclin A in Fig. 4A and B respectively were also similar, albeit each set of protein bands were turned through 180° relative to the other.

After having examined their original data, the authors realized that these figures had been inadvertently assembled incorrectly. The revised versions of Figs. 3 (showing the data correctly for the 0, 100 and 300 *μ*M/ml omeprazole experiments) and 4 (with the cyclin A data omitted) are shown on the next page. Note that the edits made to these figures do not affect the overall results and conclusions reported in the paper. The authors are grateful to the Editor of *International Journal of Oncology* for granting them the opportunity to publish this corrigendum, and all the authors agree with its publication; furthermore, they apologize to the readership of the journal for any inconvenience caused.

## Figures and Tables

**Figure 3:**
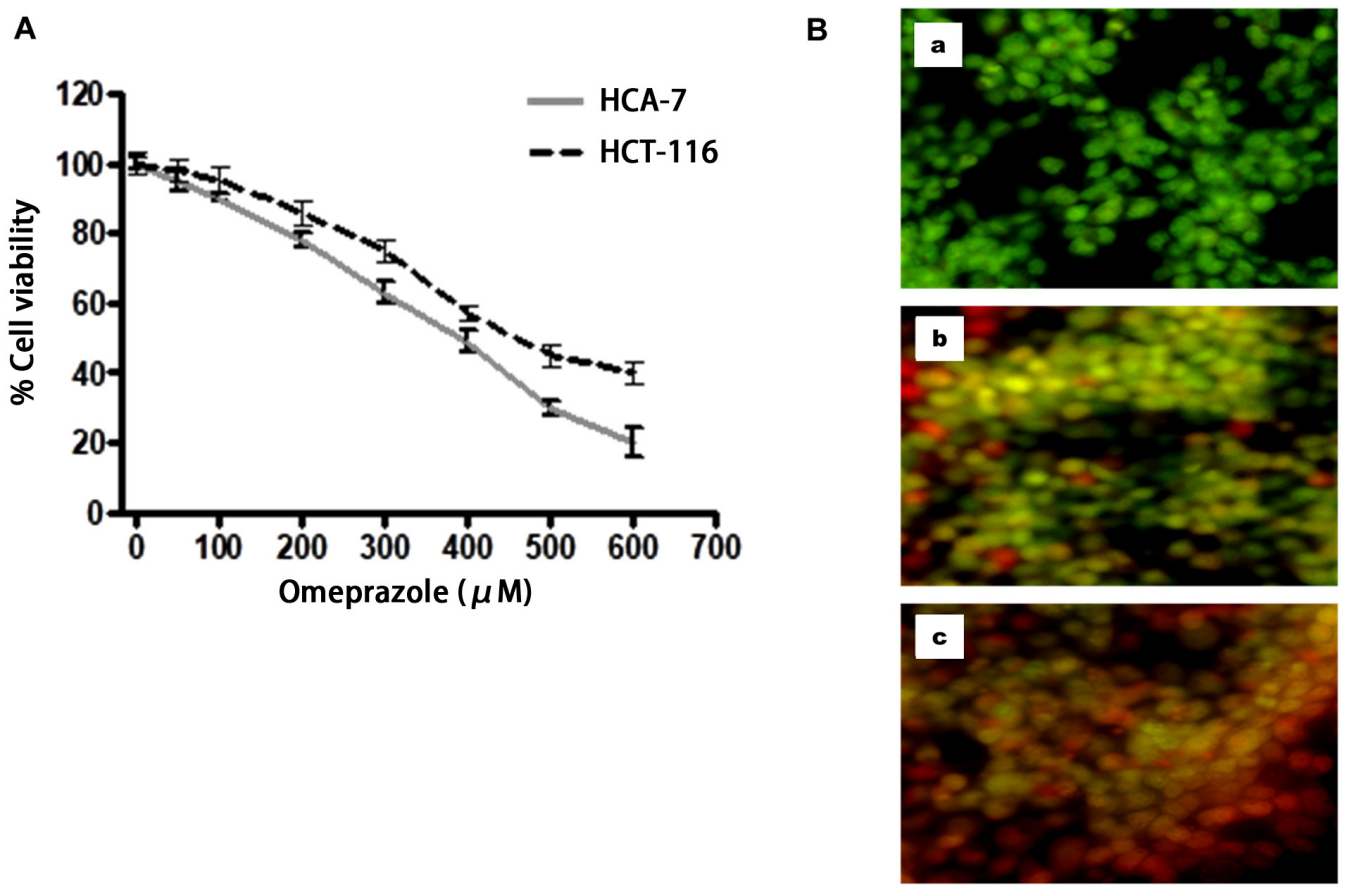
Effect of omeprazole on viability of human HCT-116 and HCA-7 colon cancer cells. (A) Cells were treated with specified concentrations of omeprazole for 24 h, and cell viability was determined by MTT assay. Each concentration of omeprazole was repeated in three wells. The values are represented as the percent viable cells where vehicle-treated cells were regarded as 100% viable. Data represent mean value of percent viable cells ± SE of three independent experiments. The details are described in Materials and methods. (B) Acridine orange/ethidium bromide staining of HCA-7 cells to detect apoptosis induced by different concentrations of omeprazole for 24 h (% apoptosis): (a) 0 μM/ml (0.2%), (b) 100 μM/ml (15%), and (c) 300 μM/ml (35%). Live cells are uniformly green, whereas apoptotic cells are characterized by orange staining due to chromatin condensation and loss of membrane integrity. Magnification, x200. Triplicate samples were used for each concentration and in each sample, a minimum of 200 cells were analyzed for apoptosis. Data are presented as % apoptosis.

**Figure 4:**
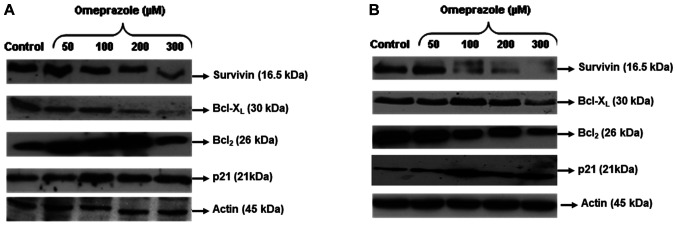
Effect of omeprazole on the protein levels of anti-apoptotic and cell cycle regulatory molecules (A) HCA-7 and (B) HCT-116 human colon cancer cells. Colon cancer cells were treated with different concentrations of omeprazole (0-300 μm/ml) for 24 h and then harvested. The protein expression of survivin, Bcl-XL, Bcl-2, and p21 were determined by Western blot analysis. Stripping the membrane and reprobing them for β-actin confirmed equal loading. The immunoblots shown are representative of three independent experiments with similar results.

